# Antenatal nephromegaly and propionic acidemia: a case report

**DOI:** 10.1186/s12882-017-0535-4

**Published:** 2017-03-30

**Authors:** Ségolène Bernheim, Georges Deschênes, Manuel Schiff, Isabelle Cussenot, Olivier Niel

**Affiliations:** 1 Pediatric Nephrology Department, Robert Debré Hospital, 48 Boulevard Sérurier, 75019 Paris, France; 2 Metabolic Diseases Department, Robert Debré Hospital, 48 Boulevard Sérurier, 75019 Paris, France; 3 Radiology Department, Robert Debré Hospital, 48 Boulevard Sérurier, 75019 Paris, France; 4 Molecular Bases of Hereditary Kidney Diseases, Inserm U1163 - Sorbonne Paris Cité - Paris Descartes University, 24 boulevard du Montparnasse, 75015 Paris, France; 5 Paris Diderot University, Paris, France

**Keywords:** Propionic acidemia, Nephromegaly, Antenatal diagnosis, Neonatal renal failure, Metabolic disease, Case report

## Abstract

**Background:**

Propionic acidemia (PA) is a rare but severe recessive autosomal disease, presenting with non specific signs in the first years of life. Prenatal diagnosis is invasive (amniocentesis) and limited to suspect cases. No screening test has been described, in particular no correlations between prenatal sonography and PA have been documented so far.

**Case presentation:**

We report the case of a boy with fetal bilateral nephromegaly and hyperechogenic kidneys, along with neonatal acute kidney injury; no etiology could be found in the first months of life. At 3 months of life, he presented with tachypnea and altered mental status, which lead to the diagnosis of PA. The renal ultrasound at 8 months of life, after a symptomatic treatment of PA had been initiated, showed a regression of the renal abnormalities.

**Conclusion:**

This case describes PA as a novel cause of large and hyperechogenic kidneys in the antenatal period. It suggests that, when confronted to fetal nephromegaly, hyperechogenic kidneys and risk factors of metabolic disease such as consanguineous parents, PA should be considered, and a prenatal test should be proposed.

## Background

Propionic acidemia (PA) is a rare but severe recessive autosomal disease caused by a deficiency of propionyl-CoA carboxylase (PCC) alpha or beta subunits, respectively encoded by *PCCA* and *PCCB*. PCC catalyzes the conversion of propionyl-CoA to methylmalonyl-CoA, upon the catabolism of valine, isoleucine, threonine, methionine, odd-chain fatty acid, and cholesterol. A PCC deficiency leads to the accumulation of metabolites of propionyl-CoA in the blood and urines, such as 3-OH-propionic acid, 3-methylcitrate, and tiglycine [1]. PA occurs in approximately 1 in 100,000 to 150,000 newborns [2]. The first clinical manifestations are non specific signs of organic acidemia (vomiting, seizures, lethargy) followed by multiple organ damages, such as development delay, cardiomyopathy, failure to thrive, liver and renal failure. Biologically, patients present metabolic acidosis with an increased anion gap, hyperammonemia, and high concentrations of propiopionyl-CoA metabolites on the gas chromatograph-mass spectrometry realized on urine. Treatment usually consists of low protein diet (8 to 12 g per day initially, up to 20 g per day), biotin and L-carnitine supplementation and intensive symptomatic care [3]. Prenatal diagnosis is invasive, as it requires an amniocentesis, and is thus limited to suspect cases. So far no correlation between prenatal sonography and PA has been described. We report the case of a boy with fetal bilateral nephromegaly associated with PA, which illustrates that renal abnormalities on routine prenatal sonography should lead to a screening of PA.

## Case Presentation

The patient was a boy, born to consanguineous parents (first degree cousins). The antenatal ultrasounds at 12 and 16 weeks of pregnancy were normal. At 24 weeks of pregnancy, the ultrasound showed bilateral nephromegaly associated with undifferentiated kidneys. Fetal weight was 467 g (45^th^ percentile). Analysis of fetal blood showed increased gamma glutamyl transferase (GGT: 2674 UI/l, normal range: 10–45 UI/l), alkaline phosphatase (330 UI/l), transaminase (ASAT: 44 UI/l), and normal beta 2 microglobulin (4,8 mg/l). The karyotype was normal: 46,XY. At 36 weeks, the ultrasound showed bilateral nephromegaly (left kidney: 66x29x33 mm; right kidney: 63x29x30 mm; normal size for the term: 43 mm) with undifferentiated kidneys. The bladder, liver, and bowel were normal. Fetal weight was 2171 g (43th percentile). Fetal biometrics were at the 20^th^ percentile. After an amnio infusion, the quantity of amniotic fluid normalized.

**Fig. 1:**
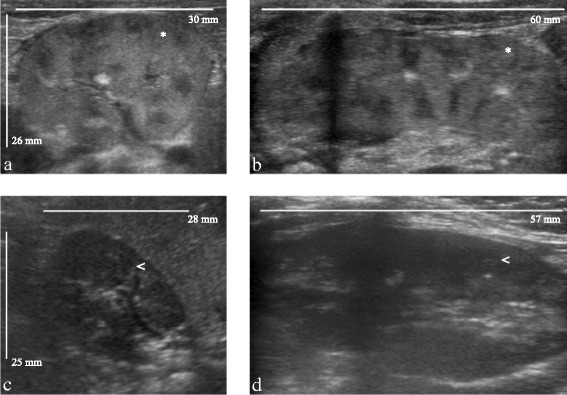
Comparison of renal ultrasounds at birth (**a**, **b**) and at the age of 8 months (**c**, **d**) in a patient with propionic acidemia. At birth, the patient presented enlarged kidneys with hyperechogenic cortexes (*). (**a**: transverse plane, **b**: longitudinal plane). At the age of 8 months, kidney size was normal, and the cortexes were normoechogenic (<). (**c**: transverse plane, **d**: longitudinal plane)

The patient was born through vaginal birth at 37 weeks and 1 day with a birth weight of 2765 g, a head circumference of 33 cm, a height of 46 cm and an APGAR score of 8 at 1 min, 9 at 5 min and 10 at 10 min. His first diuresis took place after 1 h and was 200 ml/day in the neonatal period. In the first week of life, the clinical examination was normal. Biologically he presented a cholestasis (GGT: 1110 UI/l), with a normal conjugated bilirubin (9 μmol/l). His creatinine at birth was 74 μmol/l. It decreased progressively: 69 μmol/l at 3 days of life, 47 μmol/l at 6 days of life and was normal at 9 days of life (37 μmol/l). The renal ultrasound showed bilateral nephromegaly (Fig. 1): left kidney 55x25x23 mm; right kidney 54x25x23 mm with hyperechogenic cortex, normal vascularization, and no pelvis renal dilatation. The abdominal ultrasound showed a large homogenous liver, without any dilatation of the biliary ducts. The patient was discharged after 1 week. Initial follow-up was uneventful.

At 3 months of life, the patient suddenly presented with tachypnea, followed by altered mental status (Adapted Glasgow scale according to Simon and Reilly: 6/10) and tremor of the extremities. Biological findings showed ketoacidosis (pH: 7.17; HCO3- : 12.1 mmol/L) and hyperammoniemia (181 μmol/L, normal range: 14–38 μmol/L). The chromatography of plasma aminoacids showed an increase of glycine and lysine, which led to the diagnosis of PA. This diagnosis was confirmed by genetic studies showing a mutation of *PCCB*. A whole exome sequencing was performed and showed no renal genetic development disease. The patient was treated with a low-protein diet (7.5 g per day), L-carnitine (150 mg/kg/day) and biotin (1 mg/kg/day) supplementation. Ursodesoxycholic acid (10 mg/kg/d) was introduced which led to normalization of GGT at 6 months of life (71 UI/l).

At 8 months of life, he presented no complication of PA. The patient's renal function was normal (creatinine: 14 μmol/L, Schwartz eGFR: 135 mL/min/1.73 m^2^), the ammoniemia was down to 51 μmol/L. On the renal ultrasound, the right kidney was measured at 56.7x30x28 mm, and the left kidney at 57x31x29 mm (normal size for the age: 59 mm); the kidneys were not hyperechogenic anymore. Subsequent follow-up was that of classical PA. The hepatic explorations were normal as well: ASAT: 23 UI/l; ALAT: 20 UI/l; GGT: 47 UI/l; alkaline phosphatase : 262 UI/L; bilirubin < 5 μmol/l. The hepatic and biliary ultrasounds were normal.

At 1 year and 4 months old, the renal ultrasound confirmed normal renal size (right kidney 59x27x26 mm, left kidney 62x29x27 mm), and normal echogenicity.

## Discussion and Conclusions

Hyperechogenic and large kidneys are described in different pathological groups, including renal ciliopathies. They are mainly divided into autosomal dominant and recessive polycystic kidney disease [4], glomerulocystic kidney disease (Ivermark II syndrome, trisomy 13 and 18, Beemer Syndrome), medullary cystic dysplasia (Bardet-Biedl syndrome, Meckel-Gruber syndrome, Beckwith-Wiedmann syndrome) [5], renal dysplasia, multicystic dysplastic kidneys and congenital infections (e.g., cytomegalovirus and candida). Noteworthy, in none of these diseases, a regression of the nephromegaly or of the hyperechogenic abnormality has been described, as opposed to what was observed in the patient reported here.

In some rare cases, renal failure has been described in patients with PA [6, 7]. The evolution of the patient is quite similar to the case described in [7]. Indeed, both patients presented renal failure at birth. Because of the renal failure severity, the patient in [7] required peritoneal dialysis. However both patients showed regression of the renal failure in a short time. Furthermore, in the case reported here, the ultrasound abnormalities disappeared with the normalization of the renal function. At 8 months of life, the kidneys of the patient were normal on the ultrasound, as well as his creatinine levels. In [7], peritoneal dialysis was stopped, and the patient regained normal renal function.

The pathophysiology of the renal damage caused by PA is unclear. However, renal failure due to tubulointerstitial nephritis is a known complication of methylmalonic acidemia (MMA) [2]. A study of MMA Mut −/− mice, an animal model for MMA, showed the presence of megamitochondria in renal cells of the proximal tubule [8]. Those mices developed tubulointerstitial renal disease. Furthermore, respiratory chain dysfunction, evidence of oxidative stress and megamithochondria were also found in MMA Mut −/− mice liver cells. The liver of a patient suffering from MMA was analyzed and displayed similar morphological and enzymatic finding as those observed in the murine tissue [8]. The role of oxidative stress as a result of mitochondrial dysfunction as been observed in PA [9], and could participate to the pathophysiologic process of the renal damage that was observed in the patient. Furthermore, a biopsy performed in a patient with classical PA who presented with progressive chronic kidney disease in the third decade of life showed tubular cells exhibiting focally enlarged mitochondria with disorganized cristae in electron microscopy [10]. These results suggest that a mitochondrial disease of the tubulointerstitial segment of the kidneys could account for kidney disease in PA, but need to be confirmed in other patients.

Prenatal diagnosis of PA consists of the measurement of enzyme activity or methylcitric acid in cultured amniotic fluid cells, or mutation analysis in fetal DNA [2]. Because of the risks inherent to amniocentesis, prenatal diagnosis of PA is proposed to very few families, most of them with prior metabolic diseases. Importantly, an early diagnosis of PA in neonates is associated with a lower mortality rate, even if it has no proven impact on the morbidity in survivors [11].

Our report describes PA as a novel cause of large and hyperechogenic kidneys in the antenatal period. It suggests that, when confronted to large and hyperechogenic kidneys on fetal ultrasound, once the main differential diagnoses have been ruled out, a metabolic disease such as propionic acidemia should be considered, and a prenatal test should be proposed.

## Abbreviations

MRI: Magnetic resonance imaging
PA: Propionic acidemia
PCC: propionyl-CoA carboxylase

## References

[1] Pena L, Franks J, Chapman KA, Gropman A, Ah Mew N, Chakrapani A (2012). Natural history of propionic acidemia. Mol Genet Metab.

[2] Baumgartner MR, Hörster F, Dionisi-Vici C, Haliloglu G, Karall D, Chapman KA (2014). Proposed guidelines for the diagnosis and management of methylmalonic and propionic acidemia. Orphanet J Rare Dis.

[3] Sutton VR, Chapman KA, Gropman AL, Macleod E, Stagni K, Summar ML (2012). Chronic management and health supervision of individuals with propionic acidemia. Mol Genet Metab.

[4] Emmanuelli V, Lahoche-Manucci A, Holder-Espinasse M, Devisme L, Vaast P, Dieux-Coeslier A (2012). Diagnostic anténatal des reins hyperéchogènes : à propos de 17 cas. J Gynecol Obstet Biol Reprod.

[5] Chaumoitre K, Brun M, Cassart M, Maugey-Laulom B, Eurin D, Didier F (2006). Differential diagnosis of fetal hyperechogenic cystic kidneys unrelated to renal tract anomalies: a multicenter study. Ultrasound Obstet Gynecol Off J Int Soc Ultrasound Obstet Gynecol.

[6] Lam C, Desviat LR, Perez-Cerdá C, Ugarte M, Barshop BA, Cederbaum S (2011). 45-Year-old female with propionic acidemia, renal failure, and premature ovarian failure; late complications of propionic acidemia?. Mol Genet Metab.

[7] Kasapkara ÇS, Akar M, Yürük Yıldırım ZN, Tüzün H, Kanar B, Özbek MN (2014). Severe renal failure and hyperammonemia in a newborn with propionic acidemia: effects of treatment on the clinical course. Ren Fail.

[8] Chandler RJ, Zerfas PM, Shanske S, Sloan J, Hoffmann V, Dimauro S (2009). Mitochondrial dysfunction in mut methylmalonic acidemia. FASEB J.

[9] Mc Guire PJ, Parikh A, Diaz GA (2009). Profiling of oxidative stress in patients with inborn errors of metabolism. Mol Genet Metab.

[10] Vernon HJ, Bagnasco S, Hamosh A, Sperati CJ (2013). Chronic kidney disease in an adult with propionic acidemia. JIMD Rep.

[11] Grünert SC, Müllerleile S, de Silva L, Barth M, Walter M, Walter K (2012). Propionic acidemia: neonatal versus selective metabolic screening. J Inherit Metab Dis.

